# The value of glycated hemoglobin as predictor of organ dysfunction in patients with sepsis

**DOI:** 10.1371/journal.pone.0216397

**Published:** 2019-05-06

**Authors:** Young Seok Lee, Kyung Hoon Min, Sung Yong Lee, Jae Jeong Shim, Kyung Ho Kang, Woo Hyun Cho, Doosoo Jeon, Yun Seong Kim

**Affiliations:** 1 Division of Respiratory and Critical Care Medicine, Department of Internal Medicine, Korea University Medical Center, Guro Hospital, Seoul, Republic of Korea; 2 Department of Pulmonary and Critical Care Medicine, Pusan National University Yangsan Hospital, Yangsan, Republic of Korea; University of Palermo, ITALY

## Abstract

**Background:**

In patients with sepsis, an inflammatory response can lead to destruction of the glycocalyx. These alterations cause the progression of organ dysfunction. Destruction of the glycocalyx can also occur in chronic hyperglycemia. Glycated hemoglobin (HbA1c) is a reliable marker of premorbid hyperglycemia. We investigated the association between HbA1c level at admission and the degree of organ dysfunction progression 72 hours after admission and ICU mortality.

**Methods and findings:**

This study was a retrospective observational study. Logistic regression and correlation analyses were performed to evaluate the association between the HbA1c level and the degree of organ dysfunction progression 72 hours after ICU admission. We applied survival analysis to examine the association between HbA1c level and ICU mortality. A total of 90 patients were included in this study. The association between HbA1c level and degree of organ dysfunction progression was significant (r = 0.320; P = 0.002). Multivariable logistic regression analysis showed that high HbA1c level (≥6.5%) (OR, 2.98; 95% CI, 1.033–8.567; P = 0.043) were significant, independent predictors of severe organ dysfunction progression. Patients with an HbA1c level ≥6.5% exhibited significantly greater liver and kidney dysfunction progression 72 hours after ICU admission compared with those with an HbA1c level <6.5%. Kaplan-Meier analysis showed that the survival period was significantly shorter in patients with an HbA1c level ≥6.5% than in those with an HbA1c level <6.5% (P < 0.001). Multivariable Cox proportional hazard analysis showed that HbA1c level ≥6.5% (HR, 3.49; 95% CI, 1.802–6.760; P <0.001) were significant, independent predictors of ICU mortality.

**Conclusions:**

In patients with sepsis, the HbA1c level at ICU admission is associated with progression of organ dysfunction 72 hours later and with ICU mortality. It may be important to assess HbA1c level at ICU admission because it may be a predictor of ICU outcome. For patients with a high HbA1c level (≥6.5%), greater attention should be paid to the possibility of organ dysfunction progression.

## Introduction

Sepsis is defined as life-threatening organ dysfunction caused by a dysregulated host response to infection [1, 2]. Most patients with sepsis experience progressive organ dysfunction, and death may result from multi-organ failure. Therefore, predicting and preventing organ dysfunction are key to decreasing the risk of in-hospital mortality from sepsis.

International guidelines recommend that, within the first 3 hours after hospital admission, elevated lactic acid concentration should be decreased to a normal level and mean arterial blood pressure should be maintained at 65 mmHg or higher using fluid resuscitation and vasopressors, if needed [3]. These recommendations are designed to prevent progression of organ dysfunction by correcting an abnormal macrocirculation. However, according to recent studies, organ dysfunction progression may not be prevented completely by correcting an abnormal macrocirculation because organ dysfunction progression caused by sepsis is closely related to the microcirculation [4–10].

The microcirculation plays a major role in tissue oxygenation, exchange of nutrients and waste products, modulation of inflammation, and control of coagulation. Because the microcirculation is controlled by autoregulation, microcirculatory deterioration may persist even after correcting an abnormal macrocirculation [4–10]. In patients with sepsis, increased levels of reactive oxygen species and other inflammatory mediators can lead to destruction of the glycocalyx, an important component of the microcirculation. Degradation of the glycocalyx can lead to damage of the microcirculation and contribute to or cause progression of organ dysfunction [4–14]. Therefore, predicting the extent of endothelial glycocalyx damage is important for accurately predicting the patient’s progression of organ dysfunction.

Glycated hemoglobin (HbA1c) is an indicator of the mean plasma glucose level over the past 3 months (e.g., 6% equates to 126 mg/dL; 7% to 154 mg/dL; and 8% to 183 mg/dL) [15–17]. In addition, because the HbA1c level is unaffected by the onset of critical illness, it is a reliable marker of premorbid hyperglycemia [18]. A state of continuous hyperglycemia induces endothelial glycocalyx damage, as shown in patients with sepsis [19–23]. Therefore, increased HbA1c level, which indicates chronic hyperglycemia, is related to the magnitude of baseline glycocalyx destruction, which may then facilitate the progression of organ dysfunction in patients with sepsis.

We hypothesized that HbA1c level would be associated with organ dysfunction progression and intensive care unit (ICU) mortality in patients with sepsis. We investigated the association between HbA1c level at admission and the degree of organ dysfunction progression 72 hours after admission and ICU mortality.

## Materials and methods

### Study overview

This study was a retrospective observational study performed in a cohort of patients whose HbA1c level was measured at admission to the ICU between March 2008 and August 2017 at a South Korean academic tertiary care hospital with about 1,000 beds. Among patients whose HbA1c level was measured at admission to the ICU, those who met the inclusion criteria were enrolled. We analyzed the association between the HbA1c level at admission to the ICU and the degree of organ dysfunction progression 72 hours after admission. We applied survival analysis to examine the association between HbA1c level and ICU mortality. All patients in this study received standard treatments according to the guidelines of the Surviving Sepsis Campaign [3]. All data, including clinical and laboratory data, were acquired by review of the medical records.

### Ethics statement

This study was performed in strict accordance with the principles expressed in the Declaration of Helsinki. The study was approved by the Institutional Review Board of the Korea Medical Center (IRB Number: 2018GR0266). We ensured protection of patient privacy and anonymity. The need for informed consent was waived because of the retrospective nature of the study.

### Patients

The inclusion criteria were age ≥20 years, infected patients with Sequential Organ Failure Assessment (SOFA) score ≥2 points, HbA1c level measurement at ICU admission, and direct admission to the ICU. The exclusion criteria were any condition affecting the HbA1c level (e.g., chronic kidney disease, hemolysis, other anemias, recent blood transfusion, erythropoiesis-stimulating drugs, pregnancy) [15, 24]; having signed a do-not-resuscitate order (which could bias the mortality findings); receiving cardiopulmonary resuscitation at admission; or having a severe neurodegenerative disorder that could contribute to organ dysfunction (e.g., amyotrophic lateral sclerosis, Parkinson’s disease).

### Definitions

Age and body mass index (calculated by weight divided by height squared) were included as continuous variables. Prior diagnosis of diabetes mellitus (DM) was defined as having been previously diagnosed with DM and taking noninsulin glucose-lowering agents to control DM. Causes of sepsis were based on the final laboratory, imaging, and clinical results. The laboratory findings at admission were collected. Use of vasopressors, steroids, or a ventilator, and continuous renal-replacement therapy (CRRT) were recorded only for the period of admission. ICU mortality was defined as a death during the interval from ICU admission to ICU discharge.

Sepsis-induced organ dysfunction progression was evaluated 72 hours after ICU admission for five systems (lung, cardiac, hepatic, coagulation, and kidney) using the following definitions. Lung dysfunction progression was defined as decreased partial pressure of arterial oxygen/fraction of inspired oxygen ratio as included in the SOFA score. Cardiac dysfunction progression was defined as sepsis-induced myocardial dysfunction diagnosed using echocardiography or an elevated cardiac muscle-specific creatine kinase level to more than five times the normal upper limit. Sepsis-induced myocardial dysfunction was defined as an ejection fraction (EF) <50% or a ≥10% decrease in baseline EF and return of EF to the baseline within 2 weeks [25–27]. Hepatic dysfunction progression was defined as an increased bilirubin level as included in the SOFA score. Coagulation dysfunction progression was defined as a decrease in platelet count as included in the SOFA score. Kidney dysfunction progression was defined as the need for CRRT because of acute kidney injury or an increased creatinine level as indicated in the SOFA score [28, 29].

The degree of organ dysfunction progression was calculated using a sum of dysfunction progression which was estimated at 1 score for each organ dysfunction progression. We classified the degree of organ dysfunction progression into two subsets: mild progression (score of 0–2 for organ dysfunction progression) and severe progression (score of 3–5 for organ dysfunction progression) according to the impact of the ICU mortality in this study.

### Statistical analysis

Fisher’s exact tests were used to analyze categorical data, and Mann–Whitney *U* tests were used to analyze continuous data. Correlational analysis using Spearman’s rank correlation coefficients was performed to evaluate the association between the HbA1c level at ICU admission and the degree of organ dysfunction progression at 72 hours. HbA1c levels were analyzed using receiver-operating characteristic curves to identify the cutoff levels for the optimal predictive accuracy for severe organ dysfunction progression. Survival was evaluated using a Kaplan–Meier approach and log-rank tests. Logistic regression analysis and Cox proportional hazard analysis were used to identify independent factors for severe organ dysfunction progression at 72 hours and mortality. Variables without very high intercorrelations or associations between the independent variables and having a P-value <0.1 in the univariate analysis were included in the multivariable analyses. The data are presented as the adjusted odds ratios (ORs) or hazard ratios (HRs) and 95% confidence intervals (CIs). We considered P values <0.05 in two-sided tests to indicate significance. All statistical analyses were performed using SPSS software (ver. 20.0; IBM Corp., Armonk, NY, USA).

## Results

### Clinical characteristics of patients in this study

The clinical characteristics of patients in this study are shown in Table 1. The median age of these patients was 77 years, and 49 (54.4%) were men. Their median Acute Physiology and Chronic Health Evaluation II (APACHE II) and SOFA scores at ICU admission were 24 and 9, respectively. Fifty-two (57.8%) of these patients had been previously diagnosed with DM and were taking noninsulin glucose-lowering agents to control DM. During ICU admission, no patients transferred to other hospitals.

**Table 1:** Clinical characteristics of patients in this study.

Variables	Total (N = 90)	Mild (N = 57)	Severe (N = 33)	P value
Age (years)*	77 (70–82)	77 (72–83)	77 (61–81)	0.135
Male gender	49 (54.4)	32 (56.1)	17 (51.5)	0.826
Body Mass Index (Kg/m^2^)*	20 (18–23)	20 (18–22)	21 (18–23)	0.143
APACHE II score at admission*	24 (20–28)	23 (20–28)	25 (20–31)	0.472
SOFA score at admission*	9 (7–11)	8 (7–11)	9 (7–12)	0.303
Charlson Comorbidity Index* Prior diagnosis of DM	6 (5–8) 52 (57.8)	7 (6–8) 29 (50.9)	6 (5–7) 23 (69.7)	0.164 0.121
Diagnosis				
Pneumonia sepsis	50 (55.6)	38 (66.7)	12 (36.4)	0.008
Biliary sepsis	10 (11.1)	1 (1.8)	9 (27.3)	<0.001
UTI sepsis	23 (25.6)	13 (22.8)	10 (30.3)	0.460
Other	7 (7.7)	5 (8.7)	2 (6.1)	1.000
Classification of cultured specimen				
Blood culture	35 (38.9)	17 (29.8)	18 (54.5)	0.026
Sputum culture	59 (65.6)	45 (78.9)	14 (42.4)	0.001
Urine culture	29 (32.2)	15 (26.3)	14 (42.4)	0.160
Other	3 (3.3)	0 (0)	3 (9.1)	0.046
Laboratory findings*				
C-reactive protein (mg/L)	174 (93–252)	207(104–249)	157 (76–262)	0.277
Procalcitonin (ng/mL)	7 (2–26)	4 (1–7)	11 (3–63)	0.007
Aspartate transaminase (IU/L)	44 (28–115)	39(24–64)	74 (35–360)	0.021
Alanine transaminase (IU/L)	24 (15–67)	22 (13–35)	34 (18–101)	0.036
Glucose (mg/dL)	169 (120–250)	161 (122–209)	209 (118–295)	0.148
Lactic acid (mmol/L)	4.5 (2.3–7.1)	3.7 (2.0–6.1)	5.3 (3.4–9.9)	0.006
Hemoglobin (g/dL)	11 (9–13)	11 (9–13)	11 (10–13)	0.925
Platelet (x10^9^/L)	182 (112–264)	199 (131–288)	151 (91–240)	0.038
Vasopressor use				
Norepinephrine	82 (91.1)	49 (86)	33 (100)	0.025
Vasopressin	31 (34.4)	10 (17.5)	21 (63.6)	<0.001
Dobutamine	30 (33.3)	19 (33.3)	11 (33.3)	1.000
Dopamine	27 (30)	13 (22.8)	14 (42.4)	0.060
Epinephrine	9 (10)	1 (1.8)	8 (24.2)	0.001
Steroid use	35 (38.9)	18 (31.6)	17 (51.5)	0.075
Ventilator use	76 (84.4)	48 (84.2)	28 (84.8)	1.000
CRRT use	23 (25.6)	7 (12.3)	16 (48.5)	<0.001
Ventilator days*	8 (2–14)	9 (4–18)	5 (1–11)	0.020
ICU mortality	43 (47.8)	18 (31.6)	25 (75.8)	<0.001
Glycated hemoglobin (%)*	5.8 (5.3–7.2)	5.7 (5.3–6.7)	6.6 (5.6–8.7)	0.014

Abbreviations: APACHE II, Acute Physiology and Chronic Health Evaluation II; SOFA, Sequential Organ Failure Assessment; DM, diabetes mellitus; UTI, urinary tract infection; CRRT, continuous renal replacement therapy; ICU, intensive care unit.

* Data are presented as median (25^th^ percentile-75^th^ percentile). Other variables are presented as number (percent).

At 72 hours after ICU admission, 33 of the 90 patients (36.7%) exhibited severe organ dysfunction progression. Procalcitonin, liver enzyme, and lactic acid levels were higher in patients with severe organ dysfunction progression than in those with mild organ dysfunction progression. About one-third of the patients with severe organ dysfunction progression exhibited sepsis of biliary tract origin. Patients with severe organ dysfunction progression had more severe sepsis than those with mild organ dysfunction progression because of higher percentage of those required vasopressor or steroid use. Twenty-five of the 33 patients with severe organ dysfunction progression (75.8%) died during ICU admission. HbA1c levels were significantly higher in patients with severe organ dysfunction progression than in those who had mild organ dysfunction progression (P = 0.014).

### Association between HbA1c level at admission and organ dysfunction progression at 72 hours after ICU admission

We used Spearman’s correlational analysis to analyze the association between HbA1c level at admission and the degree of organ dysfunction progression at 72 hours. The association between HbA1c level and degree of organ dysfunction progression was significant (r = 0.320; P = 0.002).

### Factors affecting the severity of organ dysfunction progression 72 hours after ICU admission

Thirty-three patients exhibited severe organ dysfunction progression during the 72 hours after ICU admission. The optimal cutoff HbA1c level to distinguish the severity of organ dysfunction progression was 6.5% (sensitivity: 54.5%, specificity: 73.7%). We used logistic regression analysis to identify the factors that predicted severe organ dysfunction progression at 72 hours after ICU admission. The univariate analysis showed that age, platelet count, levels of procalcitonin, lactic acid, glucose, and high glycated hemoglobin level (≥6.5%) were significantly associated with severe organ dysfunction progression. Multivariable logistic regression using backward elimination showed that high procalcitonin level (OR, 1.03; 95% CI, 1.005–1.047; P = 0.015), high lactic acid level (OR, 1.14; 95% CI, 1.007–1.284; P = 0.038), and high HbA1c level (≥6.5%) (OR, 2.98; 95% CI, 1.033–8.567; P = 0.043) were significant, independent predictors of severe organ dysfunction progression (Table 2).

**Table 2:** Factors affecting the severity of organ dysfunction progression at 72 hours after intensive care unit admission.

Variables	Odds ratio	95% confidence interval	P value
Univariate analysis			
Age (years)	0.96	0.920–0.996	0.031
Male gender	0.83	0.351–1.962	0.671
Body Mass Index (Kg/m^2^)	1.10	0.973–1.237	0.129
APACHE II score at admission	1.02	0.960–1.090	0.482
SOFA score at admission	1.10	0.951–1.269	0.202
Charlson Comorbidity Index	0.84	0.656–1.070	0.156
C-reactive protein (mg/L)	1.00	0.994–1.002	0.350
Procalcitonin (ng/mL)	1.03	1.008–1.045	0.005
Aspartate transaminase (IU/L)	1.00	1.000–1.001	0.315
Alanine transaminase (IU/L)	1.00	0.999–1.001	0.868
Glucose (mg/dL)	1.00	1.000–1.006	0.096
Lactic acid (mmol/L)	1.17	1.039–1.322	0.010
Hemoglobin (g/dL)	0.98	0.802–1.197	0.843
Platelet (x10^9^/L)	0.99	0.991–1.000	0.048
Glycated hemoglobin ≥ 6.5%	3.36	1.361–8.297	0.009
Multivariable analysis			
Procalcitonin (ng/mL)	1.03	1.005–1.047	0.015
Lactic acid (mmol/L)	1.14	1.007–1.284	0.038
Glycated hemoglobin ≥ 6.5%	2.98	1.033–8.567	0.043

Abbreviations: APACHE II, Acute Physiology and Chronic Health Evaluation II; SOFA, Sequential Organ Failure Assessment.

Multivariable logistic regression analysis using backward elimination was performed to investigate factors affecting the severe of organ dysfunction progression 72 hours post-ICU admission, after adjusting for 6 variables (age, procalcitonin, lactic acid, platelet, glucose and high glycated hemoglobin (≥6.5)) that were statistically significant in the univariate analysis.

### Associations between HbA1c level ≥6.5% and individual organ dysfunction progression 72 hours after ICU admission

**Fig 1:**
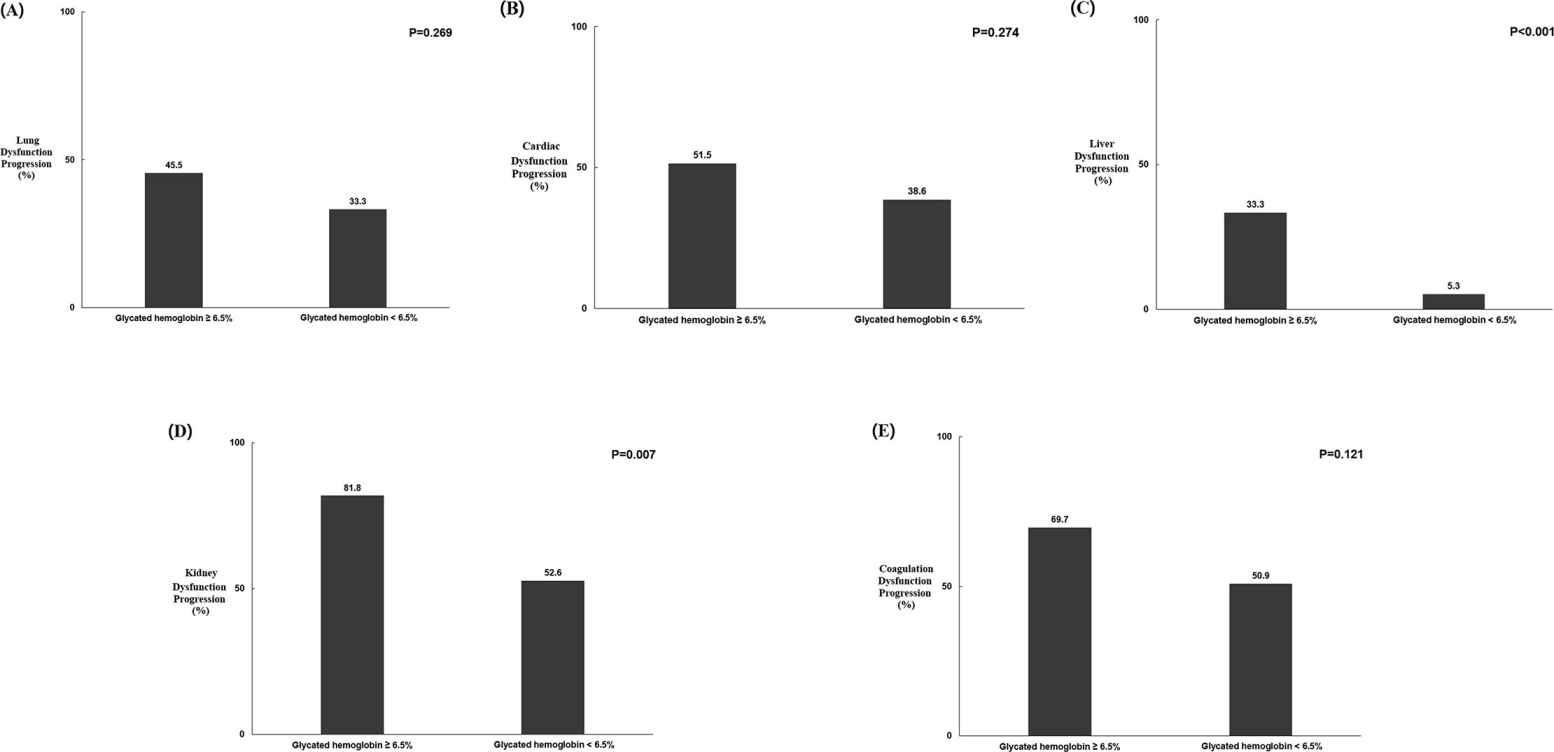
Associations between HbA1c level and individual organ dysfunction progression 72 hours after intensive care unit admission. (A) Lung dysfunction progression. (B) Cardiac dysfunction progression. (C) Liver dysfunction progression. (D) Kidney dysfunction progression. (E) Coagulation dysfunction progression.

Patients with an HbA1c level ≥6.5% exhibited significantly greater liver and kidney dysfunction progression 72 hours after ICU admission compared with those with an HbA1c level <6.5%. In addition, there was a trend for those with HbA1c level ≥6.5% to exhibit greater progression of lung, cardiac, and coagulation dysfunction compared with those with an HbA1c level <6.5% (Fig 1).

### Predictors of ICU mortality in patients with sepsis

**Fig 2:**
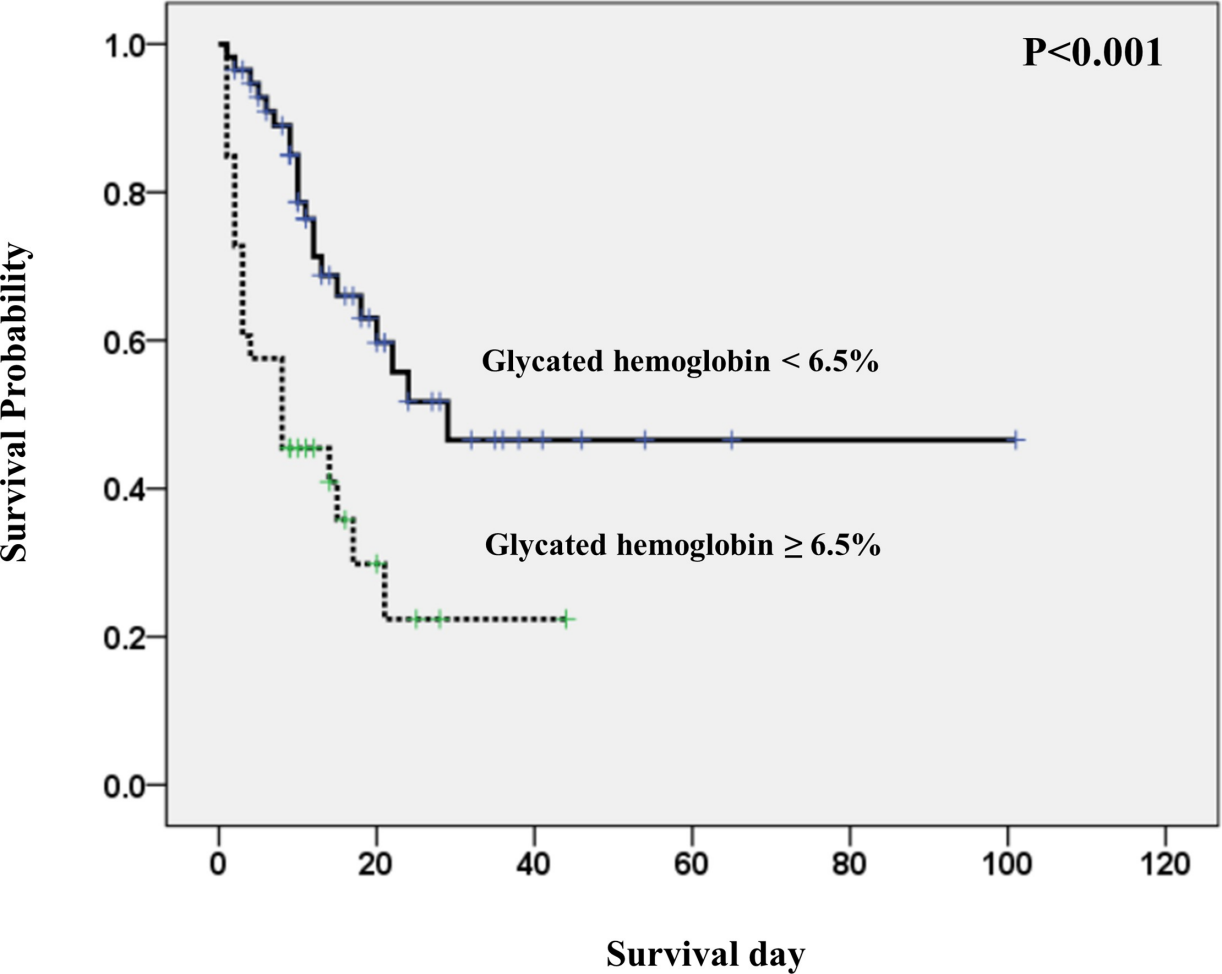
The survival curve using the Kaplan Meier method, according to high glycated hemoglobin (≥6.5%) and low glycated hemoglobin (<6.5%) (Log rank test, p<0.001).

Forty-three of the 90 patients (47.8%) died during ICU admission. Their main cause of death was multi-organ failure caused by sepsis progression. Most deaths (34 patients, 79.1%) occurred during the 2 weeks after ICU admission. We used Kaplan–Meier analysis to estimate the probability of survival during ICU admission according to the HbA1c level. Kaplan–Meier analysis along with the log-rank test showed that the survival period was significantly shorter in patients with an HbA1c level ≥6.5% than in those with an HbA1c level <6.5% (P < 0.001; Fig 2).

We then used Cox proportional hazard analysis to identify the mortality risk factors. The univariate analysis showed that an APACHE II score, SOFA score, procalcitonin level, aspartate transaminase level, alanine transaminase level, glucose level, platelet level, lactic acid level and HbA1c level ≥6.5% were all significantly associated with ICU mortality. Multivariable Cox proportional hazard analysis with backward elimination indicated that high aspartate transaminase level (HR, 1.00; 95% CI, 1.000–1.001; P = 0.007), high lactic acid level (HR, 1.07; 95% CI, 1.013–1.137; P = 0.017) and HbA1c level ≥6.5% (HR, 3.49; 95% CI, 1.802–6.760; P <0.001) were significant, independent predictors of ICU mortality (Table 3).

**Table 3:** Predictors of intensive care unit mortality in patients with sepsis.

Risk factors	Hazard ratio	95% CI	P value
Univariate analysis			
Age (years)	0.99	0.971–1.022	0.783
Male sex	1.12	0.611–2.055	0.714
Body mass index (Kg/m^2^)	0.97	0.889–1.062	0.522
APACHE II score at admission	1.06	1.011–1.108	0.016
SOFA score at admission	1.17	1.059–1.295	0.002
Charlson Comorbidity Index	0.95	0.804–1.112	0.499
C-reactive protein (mg/L)	0.99	0.996–1.001	0.272
Procalcitonin (ng/mL)	1.01	0.999–1.017	0.083
Aspartate transaminase (IU/L)	1.00	1.000–1.001	0.001
Alanine transaminase (IU/L)	1.00	1.000–1.001	0.005
Glucose (mg/dL)	1.00	1.000–1.004	0.022
Lactic acid (mmol/L)	1.08	1.022–1.134	0.006
Hemoglobin (g/dL)	1.02	0.893–1.175	0.734
Platelet (x10^9^/L)	0.99	0.994–1.000	0.054
Glycated hemoglobin ≥ 6.5%	2.79	1.524–5.120	0.001
Multivariable analysis			
Aspartate transaminase (IU/L)	1.00	1.000–1.001	0.007
Lactic acid (mmol/L)	1.07	1.013–1.137	0.017
Glycated hemoglobin ≥ 6.5%	3.49	1.802–6.760	<0.001

Abbreviations: APACHE II, Acute Physiology and Chronic Health Evaluation II; SOFA, Sequential Organ Failure Assessment.

Multivariable Cox proportional hazard analysis using backward elimination was performed to evaluate risk factors of intensive care unit mortality, after adjusting for 9 variables (APACHE II score, SOFA score, procalcitonin, aspartate transaminase, alanine transaminase, glucose, platelet, lactic acid and high glycated hemoglobin (≥6.5%)) that were statistically significant in the univariate analysis.

## Discussion

These findings support the idea that HbA1c level may be useful for predicting the degree of organ dysfunction progression 72 hours after ICU admission and ICU mortality in patients with sepsis. According to practice guidelines, patients with an HbA1c level ≥6.5% can be diagnosed with DM, and the HbA1c goal for many nonpregnant adults is <7% [15, 24]. However, our results suggest that an HbA1c level ≥6.5% increases the risk of progression of organ dysfunction and ICU mortality. Therefore, measuring the HbA1c level at ICU admission may be helpful for predicting the progression of organ dysfunction and mortality in patients with diagnosed DM and for diagnosing DM in patients with undiagnosed DM. However, it is not standard practice to measure the HbA1c level in patients with sepsis who were not previously diagnosed with DM. Our findings suggest that, when a patient with sepsis is admitted to the ICU, clinicians should consider measuring HbA1c level to help them predict the risk of organ dysfunction progression and mortality. Specifically, regardless of whether the patient has or has not been diagnosed with DM before admission, greater attention should be given to patients with a high HbA1c level (≥6.5%) because of the risk of organ dysfunction progression.

These results make several important contributions. To our knowledge, this is the first study to assess the association between HbA1c level and organ dysfunction in patients with sepsis. Although an association between the acute glycemic state and ICU outcome has been shown previously, the association between premorbid glycemic state and ICU outcome is unclear. In addition, only a few studies have shown an association between HbA1c level at admission and ICU mortality [30–33]. Our results suggest that a premorbid glycemic state predicts the degree of organ dysfunction progression 72 hours after ICU admission in patients with sepsis. For example, patients with a high HbA1c level had more severe organ dysfunction progression compared with those with a low HbA1c level.

Although the acute glycemic state may affect organ dysfunction progression during ICU admission, we believe that, for a short period of time (e.g. 72 hours after ICU admission), organ dysfunction progression is related to the premorbid glycemic state rather than to an acute glycemic state. For this reason, our study is relevant to clinical practice. However, a single parameter cannot be used to predict organ dysfunction progression in sepsis patients accurately. In this study, in addition to HbA1c level, procalcitonin and lactic acid levels were also associated with severe organ dysfunction progression after 72 hours. Therefore, several factors in addition to HbA1c level should be considered for predicting organ dysfunction progression accurately. Our findings provide support for a link between premorbid chronic hyperglycemia and progression of organ dysfunction, and suggest that HbA1c level has value as a predictor of organ dysfunction progression.

One possible explanation for these findings is that the chronic hyperglycemic state may damage the endothelial glycocalyx; this is considered to be the primary mechanism responsible for vascular complications in patients with DM [19–23]. Given that the HbA1c level reflects a patient’s premorbid glycemic state during the preceding 3 months, a high HbA1c level indicates a continuous hyperglycemic state [15]. The baseline glycocalyx damage may be more severe in patients with a high HbA1c level than in those with a low HbA1c level. The thickness of the glycocalyx in DM patients has been reported to be half of that in healthy controls [34, 35].

During sepsis, increased production of reactive oxygen species and other inflammatory mediators can also lead to glycocalyx damage [4–14]. This means that patients with a high HbA1c level who develop sepsis may experience more severe glycocalyx destruction compared with those with a low HbA1c level because of the effects of chronic hyperglycemia on the glycocalyx. From this perspective, HbA1c may not be a pathognomonic molecule in itself; rather, it reflects a chronic hyperglycemic state that may lead to baseline glycocalyx destruction.

HbA1c is a reliable marker of premorbid hyperglycemia because its level is unaffected by the onset of critical illness [18]. Degradation of the glycocalyx alters endothelial barrier permeability and may thus cause damage to the microcirculation, which contributes to organ dysfunction. Possible strategies for improving the microcirculation include performing appropriate fluid resuscitation, correcting electrolyte imbalance, maintaining glycemic control and effective insulin therapy, and considering administration of corticosteroid, albumin, and heparin or antithrombin [35]. However, further investigation of these strategies is needed to confirm whether they can improve the microcirculation and prevent or lessen the risk of organ dysfunction in patients with sepsis.

This study also revealed the most vulnerable organs in patients with sepsis and high HbA1c level. Progression of liver and kidney dysfunction was significantly higher in patients with an HbA1c level ≥6.5% at 72 hours after ICU admission (Fig 2). A previous study showed that patients with high HbA1c level are more likely to require CRRT than are those with low HbA1c level [32]. The difference in endothelial cell lining morphology and function between organs may explain the heterogeneity of organ damage seen in sepsis [4, 36–38]. The glomerular and peritubular beds in the kidney, hepatic sinusoids in the liver, and alveolar capillary barrier in the lungs are fragile under septic conditions [4, 36–38]. Our data provide evidence that the kidney and liver may be more easily injured by sepsis. However, patients with an HbA1c level ≥6.5% were more likely to exhibit progressive lung, cardiac, and coagulation dysfunction compared with those with an HbA1c level <6.5%. This issue requires further evaluation with a larger sample.

Finally, this study revealed a relationship between high HbA1c level and ICU mortality in patients with sepsis. Kaplan–Meier analysis showed that the survival period among patients was significantly shorter for patients with an HbA1c level ≥6.5% compared with those with an HbA1c level <6.5% (Fig 2). In addition, the lactic acid level and an HbA1c level ≥6.5% were significant independent predictors of ICU mortality (Table 3). We conclude that an HbA1c level ≥6.5% is associated with ICU mortality in patients with sepsis.

The present study has some limitations. First, it used a retrospective design. However, conducting a prospective study to explore this issue would be difficult because of the time and cost requirements. This study included patients with severe sepsis so that we could evaluate the relationship between HbA1c level and organ dysfunction. In total, 82 patients (91.1%) were initially injected with vasopressors because of shock, which had not been corrected by fluid resuscitation. For this reason, we were able to clarify the association between HbA1c level and organ dysfunction. Second, the total number of patients in this study (n = 90) was smaller than that in previous studies. However, measurement of HbA1c level is not routine in patients with sepsis, and we generally do not measure the HbA1c level in patients unless they have been previously diagnosed with DM. However, the total number of patients was sufficiently large to reveal an association between HbA1c level and organ dysfunction. Third, the study was not supported by experimental analysis of patients’ blood, but many previous studies nevertheless support our conclusions.

Despite some limitations, this study is the first to assess the association between HbA1c level and organ dysfunction progression in patients with sepsis. These results support the idea that HbA1c level along with other factors may be useful in clinical practice for predicting organ dysfunction progression in patients with sepsis.

## Conclusions

In patients with sepsis, the HbA1c level at ICU admission is associated with progression of organ dysfunction 72 hours later and with ICU mortality. It may be important to assess HbA1c level at ICU admission because it may be a predictor of ICU outcome. For patients with a high HbA1c level (≥6.5%), greater attention should be paid to the possibility of organ dysfunction progression. Additional prospective, multicenter studies are needed to confirm these findings.

## Supporting information

S1 Table Baseline characteristics of patients according to ICU mortality. (DOCX)

S2 Table Baseline characteristics of patients according to glycated hemoglobin level (≥6.5% vs. <6.5%). (DOCX)

S1 Fig The association between intensive care unit mortality and the degree of organ dysfunction at 72 hours after intensive care unit admission. (TIF)

## Abbreviations

HbA1c: Glycated hemoglobin
ICU: Intensive care unit
SOFA: Sequential Organ Failure Assessment
CPR: Cardiopulmonary resuscitation
DNR: Do not resuscitate
DM: Diabetes mellitus
CRRT: Continuous renal replacement therapy
EF: Ejection fraction
APACHE II: Acute Physiology And Chronic Health Evaluation II
OR: Odds ratio
HR: Hazard ratio
CI: Confidence interval
