# Supplementary material for: The value of glycated hemoglobin as predictor of organ dysfunction in patients with sepsis
Source: PLoS One. 2019 May 6;14(5):e0216397. doi: 10.1371/journal.pone.0216397 (PMC6502339; doi:10.1371/journal.pone.0216397)
Supplement: S1 Table — (DOCX) [file pone.0216397.s001.docx]

**S1 Table. Baseline characteristics of patients according to ICU mortality**

| Variables | Survival  (N=47) | Non-survival  (N=43) | P value |
| --- | --- | --- | --- |
| Age (years) * | 78 (70-82) | 76 (67-81) | 0.577 |
| Male gender | 24 (51.1) | 25 (58.1) | 0.532 |
| Body mass index* | 21 (18-23) | 20 (18-23) | 0.318 |
| APACHE II score at admission* | 22 (18-27) | 25 (21-31) | 0.059 |
| SOFA score at admission* | 8 (6-10) | 10 (7-12) | 0.019 |
| Charlson Comorbidity Index* | 7 (6-8) | 6 (5-8) | 0.251 |
| Prior diagnosis of DM | 25 (53.2) | 27 (62.8) | 0.398 |
| Diagnosis |  |  |  |
| Pneumonia sepsis | 29 (61.7) | 21 (48.8) | 0.289 |
| Biliary sepsis | 2 (4.3) | 8 (18.6) | 0.044 |
| UTI sepsis | 11 (23.4) | 12 (27.9) | 0.638 |
| Other | 5 (10.6) | 2 (4.7) | 0.438 |
| Classification of cultured specimen |  |  |  |
| Blood culture | 17 (36.2) | 18 (41.9) | 0.667 |
| Sputum culture | 35 (74.5) | 24 (55.8) | 0.078 |
| Urine culture | 13 (27.7) | 16 (37.2) | 0.373 |
| Other | 2 (4.3) | 1 (2.3) | 1.000 |
| Laboratory findings* |  |  |  |
| CRP | 203 (109-279) | 157 (84-240) | 0.234 |
| Procalcitonin | 4 (1-26) | 10 (2-24) | 0.298 |
| AST | 44 (28-64) | 44 (28-257) | 0.242 |
| ALT | 22 (13-51) | 26 (18-77) | 0.089 |
| Glucose | 156 (112-214) | 184 (132-260) | 0.105 |
| Lactic acid | 2.9 (2.2-6.1) | 5.4 (3.6-9.3) | 0.003 |
| Hemoglobin | 11 (9-13) | 11 (10-13) | 0.583 |
| Platelet | 197 (127-304) | 153 (101-237) | 0.011 |
| Vasopressor use |  |  |  |
| Norepinephrine | 39 (83) | 43 (100) | 0.006 |
| Vasopressin | 5 (10.6) | 26 (60.5) | <0.001 |
| Dobutamine | 18 (38.3) | 12 (27.9) | 0.372 |
| Dopamine | 9 (19.1) | 18 (41.9) | 0.023 |
| Epinephrine | 1 (2.1) | 8 (18.6) | 0.012 |
| Steroid use | 13 (27.7) | 22 (51.2) | 0.031 |
| Ventilator use | 38 (80.9) | 38 (88.4) | 0.391 |
| CRRT use | 4 (8.5) | 19 (44.2) | <0.001 |
| Ventilator days | 9 (5-17) | 6 (1-12) | 0.071 |
| Glycated hemoglobin ≥ 6.5% | 11 (23.4) | 22 (51.2) | 0.009 |

Abbreviations: APACHE II, Acute Physiology And Chronic Health Evaluation II; SOFA, Sequential Organ Failure Assessment; DM, diabetes mellitus; UTI, urinary tract infection; CRP, C reactive protein; AST, Aspartate transaminase; ALT, Alanine transaminase; CRRT, continuous renal replacement therapy.

* Data are presented as median (25^th^ percentile-75^th^ percentile).Other variables are presented as number (percent).
