# Supplementary material for: The value of glycated hemoglobin as predictor of organ dysfunction in patients with sepsis
Source: PLoS One. 2019 May 6;14(5):e0216397. doi: 10.1371/journal.pone.0216397 (PMC6502339; doi:10.1371/journal.pone.0216397)
Supplement: S2 Table — (DOCX) [file pone.0216397.s002.docx]

**S2 Table. Baseline characteristics of patients according to glycated hemoglobin level (≥6.5% vs. <6.5%)**

| Variables | Glycated hemoglobin | | P value |
| --- | --- | --- | --- |
|  | ≥6.5% (N=33) | <6.5% (N=57) |  |
| Age (years)* | 74 (66-81) | 78 (70-83) | 0.127 |
| Male gender | 18 (54.5) | 31 (54.4) | 1.000 |
| Body Mass Index (Kg/m^2^)* | 22 (19-24) | 20 (17-22) | 0.023 |
| APACHE II score at admission* | 27 (21-33) | 23 (19-27) | 0.015 |
| SOFA score at admission* | 9 (7-12) | 8 (7-11) | 0.099 |
| Charlson Comorbidity Index*  Prior diagnosis of DM | 6 (5-7)  26 (78.8) | 7 (6-8)  26 (45.6) | 0.028  0.004 |
| Diagnosis |  |  |  |
| Pneumonia sepsis | 17 (51.5) | 33 (57.9) | 0.661 |
| Biliary sepsis | 3 (9.1) | 7 (12.3) | 0.740 |
| UTI sepsis | 9 (27.3) | 14 (24.6) | 0.806 |
| Other | 4 (12.1) | 3 (5.3) | 0.255 |
| Classification of cultured specimen |  |  |  |
| Blood culture | 15 (45.5) | 20 (35.1) | 0.374 |
| Sputum culture | 20 (60.6) | 39 (68.4) | 0.495 |
| Urine culture | 14 (42.4) | 15 (26.3) | 0.160 |
| Other | 1 (3.0) | 2 (3.5) | 1.000 |
| Laboratory findings* |  |  |  |
| C-reactive protein (mg/L) | 200 (88-274) | 169 (95-245) | 0.791 |
| Procalcitonin (ng/mL) | 8 (3-26) | 6 (1-26) | 0.109 |
| Aspartate transaminase (IU/L) | 44 (34-159) | 42 (24-73) | 0.153 |
| Alanine transaminase (IU/L) | 35 (18-90) | 20 (13-34) | 0.011 |
| Glucose (mg/dL) | 254 (156-379) | 155 (111-194) | <0.001 |
| Lactic acid (mmol/L) | 4.9 (3.2-10.2) | 4.3 (2.2-6.5) | 0.121 |
| Hemoglobin (g/dL) | 12 (10-14) | 11 (9-13) | 0.125 |
| Platelet (x10^9^/L) | 139 (104-277) | 192 (131-266) | 0.208 |
| Vasopressor use |  |  |  |
| Norepinephrine | 31 (93.9) | 51 (89.5) | 0.705 |
| Vasopressin | 17 (51.5) | 14 (24.6) | 0.012 |
| Dobutamine | 11 (33.3) | 19 (33.3) | 1.000 |
| Dopamine | 13 (39.4) | 14 (24.6) | 0.158 |
| Epinephrine | 4 (12.1) | 5 (8.8) | 0.720 |
| Steroid use | 17 (51.5) | 18 (31.6) | 0.075 |
| Ventilator use | 29 (87.9) | 47 (82.5) | 0.561 |
| CRRT use | 11 (33.3) | 12 (21.1) | 0.218 |
| Ventilator days* | 7 (1-13) | 9 (4-16) | 0.202 |
| ICU mortality | 22 (66.7) | 21 (36.8) | 0.009 |

Abbreviations: APACHE II, Acute Physiology and Chronic Health Evaluation II; SOFA, Sequential Organ Failure Assessment; DM, diabetes mellitus; UTI, urinary tract infection; CRRT, continuous renal replacement therapy; ICU, intensive care unit.

* Data are presented as median (25^th^ percentile-75^th^ percentile). Other variables are presented as number (percent).
