# Supplementary figures and images for: The value of glycated hemoglobin as predictor of organ dysfunction in patients with sepsis
Source: PLoS One. 2019 May 6;14(5):e0216397. doi: 10.1371/journal.pone.0216397 (PMC6502339; doi:10.1371/journal.pone.0216397)

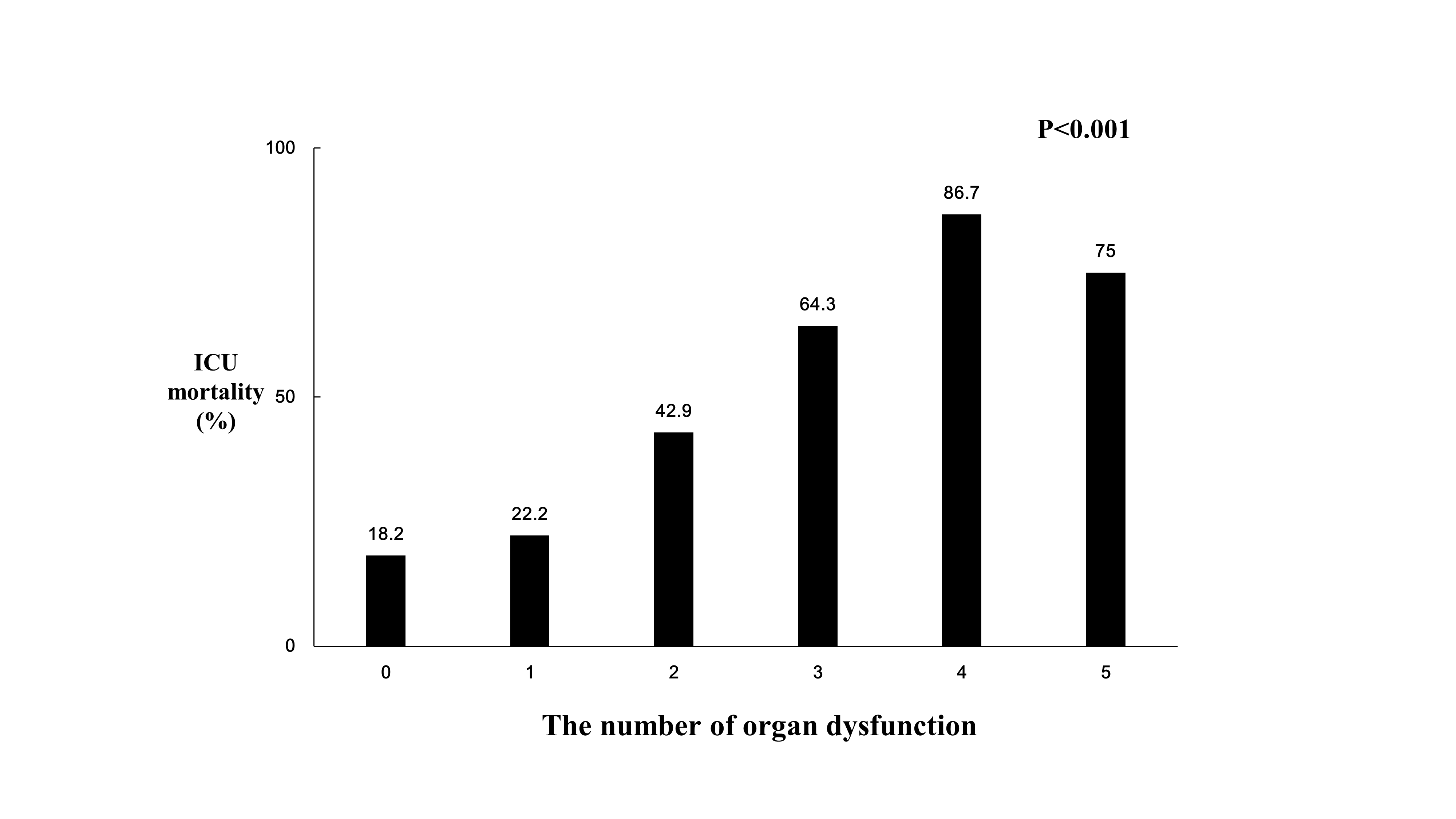

Supplement: S1 Fig — (TIF) [file pone.0216397.s003.tif]
